# World-beating? Testing Britain's Covid response and tracing the explanation

**DOI:** 10.1017/S174413312000033X

**Published:** 2020-08-19

**Authors:** Calum Paton

**Affiliations:** Public Policy, Keele University, Keele, Staffordshire ST5 5BG, UK

**Keywords:** Covid-19, Johnson government, policy failure, UK

## Abstract

The UK, and England in particular, has suffered egregiously poor outcomes in managing the Covid-19 pandemic. This short perspective points to the explanation in terms of both current British politics and the public health policy inheritance. Boris Johnson's Premiership was born in an opportunistic assertion of British exceptionalism, and Johnson's initial, fate-tempting reaction to the novel Coronavirus set the UK on the wrong path. Furthermore, the gradual erosion of professionalism in (especially health) policy-making over almost four decades, and the hollowing-out of the health protection infrastructure, both facilitated and accentuated a toxic approach to managing Covid-19.

Political science qua science does not explain much about how different countries have had different success and failure rates in dealing with Covid-19. Countries with very different political structures, regimes and governments (and combinations thereof) have acted similarly – some effectively, producing good outcomes; some badly, with poor outcomes. Conversely, politically similar countries have produced a diversity of responses and outcomes. It is argued here that the UK's policy failures on Covid-19 (Grey *et al*., 2020; Mueller and Bradley, 2020), and the extent to which the UK has been an ‘outlier’ in its approach, reflect the distinctive nature of the Johnson government, enabled by the inherited public health policy structure and culture.

## 1. Criteria for failure, causes of failure

The UK has produced an egregiously poor outcome, the worst in Europe and one of the worst in the world, whether one measures death rates (per population) by numbers who have died following a positive test for Covid-19, numbers of deaths with Covid as the cause on the death certificate or excess numbers of deaths by reference to a previous five-year average, at the time of writing.

Admittedly there are other outcomes from the crisis which may be considered salient as well as Covid-caused deaths, although many would rate the latter as the real and present danger. For example, even focussing solely upon public health, it is argued that economic crisis has its own health consequences, through factors such as unemployment and isolation. A lockdown of society, specifically, may have some negative consequences for health as well as the obvious positive effect of saving lives and averting serious morbidity from Covid. Nevertheless, it is plausible that an effective suppression of Covid, even with a drastic short-term economic down-turn, may allow more sustainable long-term recovery.

What is more, the ‘knock on’ health effects of a lockdown are not all negative. Less environmental pollution and fewer road traffic accidents are but two examples. While a lockdown is of course an inevitably short-term strategy to suppress Covid, this does not invalidate the point. The unsustainability of the prevailing economic model in the context of global warming may be reinforced in public debate as a result of a graphic illustration of how pollution, for example, can be diminished. Simply restoring the status-quo ante after lockdown is a missed opportunity. It is therefore important to recognise that a concern for economic welfare is not a simple, bi-polar opposite to a concern for suppression of Covid. Furthermore, governments which wish to prioritise economic activity and ‘normality’ over the fight against Covid may present worries about (unquantified) negative health effects of a lockdown, for example, as a smokescreen for their real concerns.

Within the UK, England has demonstrated significantly worse statistics than (especially) Scotland and the other home countries as time has passed, from March to July 2020, in terms of both death and infection rates. The UK government is de facto the English government for health policy, as the other three UK nations have devolved responsibility for health. I will argue below that the UK government has been a laggard at every significant stage of potential control of the virus, by comparison with those countries globally which have significantly suppressed Covid. In this context, its strikingly poor outcomes are not surprising.

What do we mean by too little, too late? Unlike countries as politically diverse as New Zealand (a Westminster-style liberal democracy), South Korea and Taiwan (new democracies) and various South East Asian authoritarian regimes from Vietnam to Hong Kong, the UK failed to suspend flights from Corona hotspots. This made effective quarantine of arrivals necessary as the next best step. The UK did not do this, apart from one or two token flight arrivals.

The next step therefore had to be ‘track and trace’ the virus from the outset. But any competent executive would have known already that the UK's capacity to test and trace had been decimated. A Cabinet Office report in 2015, Exercise Cygnus, set out the extent of government spending cuts in health protection and how warnings about lack of preparedness for control of pandemics had been buried (Cabinet Office, 2015). So speedy initial action on flights and quarantine had been all the more necessary. Worse, when the creation of a viable new test and trace policy became vital, the UK government's new test and trace scheme took months to develop. It was heralded as ‘world-beating’, but, as the New York Times noticed, the British government was better at self-congratulation than delivery (Mueller and Bradley, op. cit.).

When in March an earlier, limited attempt to track and trace Covid cases had to be abandoned through lack of capacity, this decision was presented as a scientific decision based on the fact that Covid-19 was now endemic within the population. But a failure to be blunt with the public was arguably not helpful in maintaining popular trust later on, and therefore more effective popular compliance with difficult measures to come. Previous administrations, not the Johnson government, had stripped out Britain's public health protection capacity. But, if testing therefore had to be abandoned, then an immediate, rigorous lockdown was logically required. There was a substantial risk of widespread community infection having taken place, even in the absence of hard data at that point. This is by no means a question of hindsight bias. Yet equivocation and delay occurred on the basis that ‘the science’ did not yet justify a lockdown. It seemed odd that there were too many cases to allow community testing to be possible, yet also too few to justify lockdown.

Evidence that the government, advised and supported by its Chief Scientific Officer and Chief Medical Adviser, was indeed pursuing the disastrous idea of ‘herd immunity’ has now come to light (Titheradge and Kirkland, 2020). While conceivably applicable to a non-lethal influenza pandemic (the basis for much of the government's modelling orthodoxy), this approach was eminently unsuitable for Covid-19.

Reliance on herd immunity might be an alternative explanation to incompetence as the reason for neither ‘test and trace’ nor a lockdown happening before they did, but it is hardly a reassuring one. In any case, the fact that Imperial College's modelling (Ferguson *et al*., 2020), on the one hand, and an alleged late-night phone call from a frustrated French President (Channel 4 TV, 2020) on the other, seem to have persuaded the Prime Minister to change tack, reluctantly but abruptly, suggest that the government's approach was unsure and unstable.

Even if we were to accept the government's denial that herd immunity was a policy as opposed to a prediction, at all four possible decision-points on a critical path to controlling the virus (suspension of flights; early quarantine; test and trace at the outset; lockdown), the UK/English government either did nothing or acted too late in too limited a manner. We might also consider that the English lockdown was modest in relative terms, although in absolute terms it inevitably seemed draconian; that it was undermined by mixed messages; and also poorly policed.

It may also have been ended too soon even by the government's own criteria comprising levels of threat and conditions for easing lockdown. For example, the government had devised, for England, five ‘Levels’ (Cabinet Office, 2020) of what were in effect Covid incidence and prevalence, with different relaxations of the lockdown associated with a move in the right direction from a higher to lower level. Yet the government announced on 30 May the re-opening of retail (a Level 3 development) when the country was still at Level 4, and also announced the recommencement of certain sports events and the relaxation of the rules for those vulnerable people ‘shielding’ themselves (Level 1 developments) when the country was at Level 3.

It was announced on 23 June that the measure of social distancing was to be reduced from 2 m to ‘one metre plus’ in readiness for shops (followed by pubs and restaurants) re-opening and a return of most employees to work, despite the government's own scientific advice that the risk of 1 m was up to ten times the risk of 2 m (Sage, 2020). Under pressure from its own backbenchers to ‘open up’ despite relatively high levels of risk, it was possible that the government was seeking to ‘normalise’ for England what were – by European (and other UK country) standards – high levels of infection and death.

## 2. Brexit and the Boris factor

The Johnson government was born in Brexit. Jumping on the Brexit bandwagon was what made Johnson Prime Minister. ‘Getting Brexit done’ was what legitimised his clear-out of the upper echelons of the Conservative Party, in preparation for his election victory in December 2019, which in turn meant that his post-election Cabinet was notably loyalist. On 31 January as the UK formally left the EU, Johnson's political strategy combined national pride with globalist economic policy.

Only three days later, on 3 February, he made a classic hostage-to-fortune speech which set the UK on the wrong path as regards the new Coronavirus (Johnson, 2020). Britain was to be the world leader shedding its ‘Clark Kent spectacles’ to become the national Superman resisting an exaggerated response to public health threats, in the interest of avoiding ‘market segmentation’. Post-Brexit Britain could not afford, in more ways than one, to be blown off course. But this stymied effective action on Covid, not logically but psychologically. Admittedly there was the institutional memory that over-reaction to the swine flu threat had cost billions in the recent past. Yet modelling future threats from pandemics too restrictively, in terms of influenza, now retarded timely action against Covid, and that is by no means only a judgement with the benefit of hindsight (Grey and MacAskill, 2020).

This does not mean that Brexit was right or wrong. It simply suggests that a government such as Johnson's, when one adds in the Prime Minister's self-image as an optimistic risk-taker in the Churchillian mould, sought to reconcile its Covid response with its overall narrative of optimism. The new Britain was to be a ‘global, trail-blazing’ country (Binding and McGuinness, 2020). Johnson, even on 19 March (less than four days before doing a volte-face and announcing a formal lockdown), promised to ‘send Coronavirus packing’ and gave the timeframe as ‘twelve weeks’ (Prime Ministerial Briefing, 2020). On 3 March, Johnson had announced (Downing Street Press Conference, 2020), ‘I was at a hospital the other night where I think a few there were actually coronavirus patients. And I shook hands with everybody, you'll be pleased to know, and I continue to shake hands.’ Johnson shook hands with both presenters on ITV's This Morning on 5 March, and at Westminster Abbey on 9 March.

This was despite a document produced by SPI-B, the government's pandemic advisory group on behaviours, on 3 March stating, ‘there was agreement that government should advise against greetings such as shaking hands and hugging, given existing evidence about the importance of hand hygiene’ (SPI-B, 2020). The Prime Minister was either being deliberately ‘exceptionalist’ in his approach to Covid or was simply unaware of key advice from within his own government. Either way, it was a loose approach by comparison with those countries which achieved significantly better outcomes than Britain. Quite literally, his body language undermined the seriousness of the scientific case.

Unfortunately, events were to show that Britain's only claim to ‘trail-blazing’ lay in Covid death figures. Failure to prepare against an external enemy (an invisible one, in Johnson's metaphor) sadly savoured less of Churchill, Johnson's hero (Johnson, 2014), than of his predecessor as Prime Minister, Chamberlain, at the beginning of World War II. Whether intentionally or not, some of the government's lockdown measures were undermined by equivocal and mixed messages from Johnson himself and some Ministers. The changing of the official strapline from ‘Stay at Home, Save Lives’ to ‘Stay Alert, Save Lives’ on 10 May in England but not the rest of the UK (despite the fact that, by this time, the rest of the UK was doing better than England) is but one example. (Later, to give another example from the many, when it seemed that the British public was misinterpreting the incremental loosening of lockdown, highlighted by scenes of crowded beaches on 25 June, the Prime Minister was silent and, unusually, Chief Medical Officer for England Chris Whitty tweeted his anxiety the following day.) Perhaps Johnson feared being seen as a kill-joy as well as, in Trumpian style, fearing a legacy of economic failure, but mixed messaging was heavily criticised by scientific experts (Science Media Centre, 2020).

## 3. Sofa government and the subjugation of the health apparatus

From the 1980s onwards, a succession of governments both Conservative and Labour were preoccupied with continual reform of the Department of Health and the governance of the NHS and – usually as an after-thought to such reform – structural change to the public health apparatus weakened it, whether intentionally or not. Motivation for reform varied from a frustration with ‘the forces of conservatism’ in the bureaucracy to a more venal desire to control and co-opt any challenge to the government's agenda.

The Thatcher governments began the process; subsequently, the Blair governments deepened the process of NHS reform (Paton, 2016). Little noticed but significant was the merging of the roles of NHS Chief Executive (CE) and Permanent Secretary (PS) to the Department of Health in 2000. This and other changes led to the DoH becoming known as the ‘Department of Delivery’ (Greer and Jarman, 2007). But if the policy ‘deliverable’ is suspect, then ‘managerialising’ and co-opting independent sources of advice will have harmful effects. The later re-separation of the roles of PS and CE in 2006 did not restore the salience of ‘mandarin’ (traditional senior civil service) advice to Ministers. It simply reduced the influence of the traditional civil service. The Johnson government moreover clipped the wings of Departments which were not willing to tie themselves to the chariot wheels of over-powerful centrally approved political advisors led by Dominic Cummings, Johnson's chief adviser.

Sofa government – which I would define as excessively informal government with excessive attention to political advisers' whims and ideological wheezes – had its apotheosis in the Health and Social Care Act of 2012 which dis-integrated both the NHS and the public health and the public health apparatus. The resulting structure (and culture) made it easier for the government to take its distinctive approach to Covid.

The competence and integrity of Ministers and partisan appointees to crucial ‘Covid management’ roles soon became a cause for worry. For example, Home Secretary Priti Patel could not give even basic facts concerning the belated quarantine policy to the House of Commons Select Committee on Home Affairs (2015). Baroness Dido Harding (Number 10 Downing Street Covid-19 Briefing, 2020), the Conservative peer chosen to chair the new Test and Trace Authority, gave information found to be false by BBC fact-checking. She claimed that test and trace had improved its performance week on week, when in fact its current ability to trace contacts of those infected had fallen by 20% from a month previously. This took it below the threshold at which it can be effective, according to the government's own Scientific Advisory Group on Emergencies (Sage). Sage itself was under challenge for being too politically compliant, by former Chief Scientific Adviser Sir David King, who constituted an ‘Independent Sage’.

News management more generally became a concern. To give a few examples: on 18 July, Health Secretary Matt Hancock suspended the publication of data giving daily deaths from Covid of those who had been tested, on the grounds that it over-estimated the number. Unlike in the rest of the UK, there was no cut-off point of 28 days between test and death beyond which the death was not attributed to Covid. This made a marginal change. Assuming that a later death is not attributable to Covid seems dubious. But more importantly, Hancock was silent about more accurate data still – that which showed the actual death rate to be up to 20,000 higher than the official tally, at 65,000 by mid-July. Even if one took deaths mentioning Covid on the death certificate as the main cause of death (whether or not the patient had been tested), the number was 55,000 as opposed to the officially provided 45,000.

In order to obscure the true rate of community infection, the government had refused – until a row in Parliament – to release its own centrally collected ‘Pillar 2’ data giving positive Covid tests outside hospitals and care homes, in the process hamstringing local government's ability to take feasible local action to control the spread of the virus (BBC News, 2020). On 20 July, Ruth May, the (UK/English) Department of Health's Chief Nurse, confirmed to the House of Commons Public Accounts Committee (2020), that she had been withdrawn at short notice from a daily No. 10 Downing Street Covid-19 briefing for asserting, in the ‘ preparation session’, that, if questioned about the controversial breaking of the lockdown by Dominic Cummings, the Prime Minister's chief adviser, she would answer that the law applied to everyone.

## 4. The controversial role of official scientific expertise

Because of the hollowing out of England's public health structure, the new test and trace structure was developed nationally, ignoring local expertise and relying on private companies (some with a dubious record of success with previous government contracts) to deliver, using previously unskilled staff on near-minimum wage. Only belatedly was local government given responsibility for local outbreaks of infection, and only in mid-July did local Directors of Public Health receive data about their own localities at postcode level – which, if received weeks or months earlier, could have enabled prevention of spread more cogently than the still-underperforming national ‘test and trace’ programme (BBC, 2020).

Arguably, a structural change had weakened the public health apparatus: while the 2012 Health and Social Care Act had returned responsibility for local Directors of Public Health to local government rather than the NHS, they were now also to be responsible ‘upwards’ to Public Health England, under tight government control by comparison with its predecessor agencies. As with so many health reforms, the rhetoric of the 2012 Act had been devolution, yet the reality increased central control. To give one example, on August 13, Public Health England yielded to Ministerial pressure to prune Covid mortality figures. Its headline reported figure became ‘deaths within 28 days of a positive Covid test', a restrictive figure especially in the light of increasingly available available partial treatments which might save lives but also, in other cases, delay death. As mortality fell, the proportion of deaths later than 28 days, such as deaths within 60 days or with Covid as cause of death on the death certificate, grew in salience. If Health Secretary Matt Hancock were serious about accuracy, deaths certified as Covid deaths (with or without a test) or overall excess mortality would fit the bill, not a pruning of an already restrictive measure.

The government made much play initially of ‘following the science’ – that is, trusting the professional experts – although later the slogan was changed to ‘listening to the science’. Whichever, leading scientific advisers did not help their cause by seemingly interpreting ‘science’ as waiting for enough data to demonstrate cause and effect. This is faintly ludicrous: how can one wait for a disaster to hit, on the grounds that acting to prevent it would have to be done in the absence of data about disastrous outcomes?

For example, it is claimed by the government that lack of information (or wrong information) about the rate of spread of the virus meant that instigating an earlier lockdown was not justified by the data. Yet the most cursory awareness of the situation in Italy, France and perhaps Spain, which were ‘ahead’ of the UK in terms of Covid's arrival and spread, would have disabused any decision-maker of the notion that having a lockdown too soon was damaging.

At the time, Chief Medical Adviser Chris Whitty (in the Number 10 Briefing, 5pm, 12 March 2020) made an implicit appeal to behavioural science in claiming that ‘too soon’ a lockdown might be unacceptable to the population if it led by to lockdown ‘fatigue’. Yet this message was opposed in an open letter to the government from hundreds of behavioural scientists claiming that there was no such evidence from their discipline, although there was different evidence which could help the government. (See Lunn, 2020, and Oliver, 2020, for clarification of such issues.) It is difficult to imagine that there could in fact have been such evidence from any event or circumstances similar to such a novel pandemic.

Other examples of controversial decisions backing a looser approach to Covid exist, granted that the definitive evidence may not always be conclusive. Deputy Chief Medical Officer Jenny Harries has attracted retrospective attention for her claim that large sports events were not risky (in a video interview with Boris Johnson at No. 10 Downing Street, 11 March), only one week before the initial ‘voluntary’ lockdown was announced on March. Britain and England specifically, was also late to the table in mandating the wearing of masks in various public places, and (once again) did so with a light touch in enforcement. The evidence may be contested, but if one accepts the precautionary principle, it is reasonable to interpret the emerging evidence from the UK (for example, the high rate of infection among healthcare staff in the UK where there were shortages of masks compared to settings where there were not) as a reason for action (Greenhalgh *et al.*, 2020).

Perhaps most worryingly of all, those who provided the ‘science’ to be followed seemed to be flirting with a version of science which was not only controversial but dangerous in the wrong hands. Herd immunity, discussed above, was identified publicly with Chief Scientific Adviser Patrick Vallance following his interview on the BBC Radio 4 Today programme on 12 March. It would not be fanciful to assume that, to a ‘trail-blazing’ government such as Boris Johnson's, a ‘science’ which obviated the need to lockdown or take other drastic measures would be attractive. Herd immunity was believed to be possible at this stage, to be fair – but only achievable over a long time period and at the cost of many deaths ‘upfront’.

Even believing in its achievability in the absence of a vaccine is a stretch, however, particularly in the light of later data: 7% of the UK population (Office for National Statistics, 2020) and 5% of the Spanish populations (Pollán *et al*., 2020) were estimated to have been infected after the peak of (the first wave of) the pandemic. These figures meant significant numbers of lives at risk, with Britain's excessive death toll reaching 65,000, but nothing remotely near the numbers required for herd immunity, which required anything from 60 to 80% of the population to be infected. Furthermore, in the absence of information about whether having had the illness prevents reinfection, advocating herd immunity, or even considering it seriously, seems (not only in hindsight) to be a recipe for damaging delay in taking the necessary measures.

Whether the scientific experts were going along with political necessity or unwittingly sending the government down a fateful path, time may tell. Meanwhile we may note that Prime Minister Johnson – it was claimed by the Italian Health Minister (Channel 4 TV, op.cit) – had told the Italian Prime Minister that ‘we are going for herd immunity’.

It was a risk-taking government's misfortune that, no sooner elected, it was confronted with a crisis which required risk-aversion. But that does not justify a failure to act responsibly. Longer-term political self-interest might also have given Johnson pause. Opinion polls were by July 2020 showing a clear majority of Scots in favour of Scottish independence, more than ever before at 54%, largely as a result of how Covid had been handled, with Scots giving their devolved Premier, Scottish Nationalist Party First Minister Nicola Sturgeon, a 60% approval rating and Boris Johnson, the UK Premier, a ‘minus 39%’ (dis)approval rating (Curtice, 2020). What an irony if the break-up of the UK was Boris Johnson's Covid legacy.
